# Benchmarking intermediate dose spillage in single target stereotactic radiosurgery for brain metastases

**DOI:** 10.1016/j.phro.2026.101105

**Published:** 2026-10-09

**Authors:** Youness Nour, Shari Wiegreffe, Tuba Tekin, Lara Caglayan, Christina Leitzen, Cas S. Dejonckheere, Davide Scafa, Julian P. Layer, Victoria Volkenborn, Fabian Kugel, Stephan Garbe, Christos Moustakis, Dimos Baltas, Gustavo R. Sarria, Anca-L. Grosu, Eleni Gkika

**Affiliations:** a Department of Radiation Oncology, University Hospital Bonn, Venusberg-Campus 1, Bonn, Germany; b University Clinic for Medical Radiation Physics, Medical Campus Pius Hospital, Carl von Ossietzky University, Oldenburg, Germany; c Institute of Experimental Oncology, University Hospital Bonn, Bonn, Germany; d Department of Radiation Physics, University Hospital Leipzig, Stephanstrasse 9a, Leipzig, Germany; e Department of Radiation Oncology, University Medical Center Freiburg, Faculty of Medicine, University of Freiburg, Freiburg, Germany

**Keywords:** Stereotactic radiosurgery, Brain metastases, Intermediate-dose spillage, Quality assurance, Dose gradient

## Abstract

**Background and purpose:**

Intermediate-dose spillage is central to normal-brain sparing in stereotactic radiosurgery, yet fixed adverse-event and relative indices do not define achievable plan quality for single-target linear accelerator treatments. The aim was to derive a volume-dependent absolute benchmark for the 50% prescription isodose volume (V_50%_; equivalent to V_10 Gy_ for 20 Gy plans) for brain metastases.

**Materials and methods:**

In total, 276 single-isocenter single-target plans were analyzed: 178 idealized model plans and 98 clinical plans. Power-law regression in log-log space modeled spillage against planning target volume (PTV, cm^3^). The volume-scaling exponents were 0.83 for the ideal references and 0.75 for the simplified clinical threshold. The clinical upper threshold was derived using q = 0.90 quantile regression. Threshold-exceeding plans were reviewed for anatomical or technical justification.

**Results:**

In the model cohort V_50%_ value correlated strongly with PTV (*r* = 0.994, *p* < 0.001). The ideal mean model was V_50%_ = 3.71 × PTV^0.83^, the conservative ideal-planning reference was V_50%_ = 4.10 × PTV^0.83^, and the clinical upper threshold was V_50%_ = 5.00 × PTV^0.75^. Ten of 98 clinical plans (10%) exceeded the threshold. Seven lacked anatomical justification and were replanned, reducing V_50%_ by a median of 14% (range 7–29%) without compromising target coverage.

**Conclusions:**

A volume-based V_50%_ benchmark provided a complementary quality-assurance reference for single-target linear accelerator radiosurgery for brain metastases. It flagged plans that warranted structured review alongside target coverage, organs of interest, conformity, and gradient metrics.

## Highlights

• A 10 Gy threshold was derived from 276 radiosurgery plans for brain metastases.
• Target volume explained 99% of 10 Gy spillage in idealized plans.
• The threshold identified 10 of 98 clinical plans needing structured review.
• Replanning reduced 10 Gy spillage by a median 14% in seven plans.

## Graphical abstract

**Figure:**
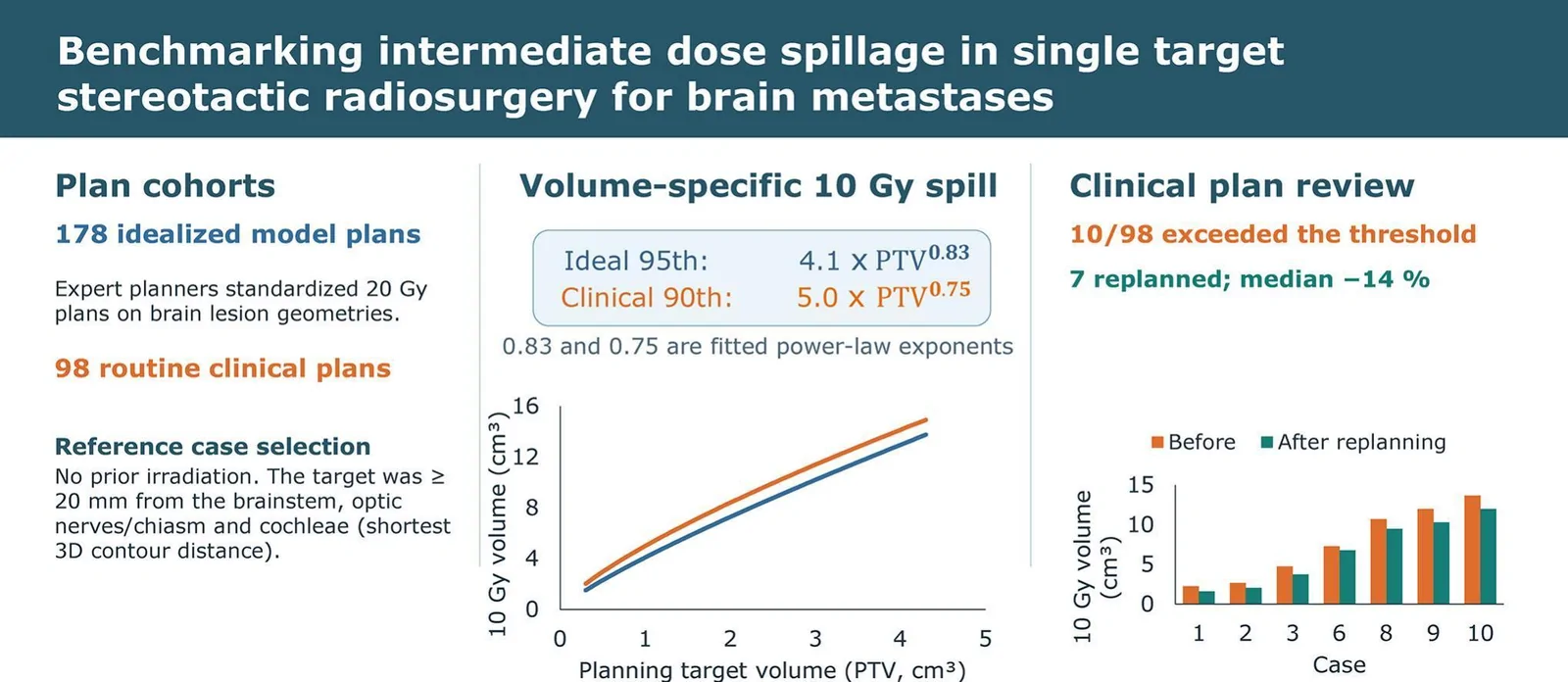

## 1 Introduction

Stereotactic radiosurgery (SRS) is a well-established modality for the management of brain metastases, characterized by precise targeting and steep dose gradients aimed at minimizing dose exposure to surrounding healthy tissue [1], [2], [3], [4]. With the increasing incidence of brain metastases and prolonged survival due to advances in systemic therapies, the clinical demand for efficient, reproducible, and safe SRS techniques has grown substantially [5].

Importantly, many patients return for additional salvage SRS as new lesions emerge over time; therefore, a plan that remains below fixed dose-volume limits at first treatment may still deliver avoidable intermediate-dose exposure and limit the feasibility of subsequent SRS courses, particularly in the reirradiation setting.

In linear accelerator-based SRS, optimizing dose fall-off is critical. Historical guidelines, derived from studies of adverse events, established fixed thresholds for intermediate-dose spillage (e.g., V_12Gy_< 8.5 cm^3^ or 10 cm^3^) to predict radionecrosis risk [6], [7]. However, fixed adverse-events limits alone are insufficient for plan-quality assessment. For small lesions, a plan with 9 cm^3^ of spillage for a 0.5 cm^3^ target could meet such limits while still exceeding the dose fall-off that is physically achievable. Conversely, strictly applying these limits to large targets may be physically impossible.

Therefore, a shift is needed from binary adverse-event thresholds to continuous, volume-dependent benchmarks that define the limit of optimality rather than just the limit of safety. While existing prediction models attempt to address this, they predominantly originate from robotic radiosurgery systems or are limited to categorical metrics such as the gradient index (GI) or R _%_, the ratio of the 50% prescription isodose volume to the planning target volume [8], [9], [10], [11], [12].

Recent studies have described power-law relationships for GI and R_50%_ in single-isocenter multiple-target (SIMT) settings [13], [14]. It remains unclear whether these relationships, while driven by the geometric surface-area effect, are universally transferable to single-isocenter single-target SRS (SIST), where confounding factors such as inter-target dose modulation are absent. Establishing a single-target baseline is also a prerequisite for quantifying the additional spillage that may be accepted when SIMT is used.

The aim of this study was therefore to validate these volume-scaling relationships in a large independent cohort of single-target linear accelerator SRS plans for brain metastases. By translating relative indices into absolute volumetric metrics (V_50%_), a volume-dependent prediction model and a clinically actionable upper threshold for routine plan review were derived.

## 2 Materials and methods

### 2.1 Study design and cohort definition

This study was approved by the Ethics Committee of the Medical Faculty of the University of Bonn (approval no. 2024–105-BO). To assess volume-based models for intermediate-dose spillage in SRS, a total of 276 datasets were analyzed in a two-phase approach. Planning objectives and dose guidance are summarized in Supplementary Table S1.

For model generation, 178 idealized treatment plans were simulated based on lesion geometries meeting specific criteria: planning target volume (PTV) 0.3–4.3 cm^3^, no prior irradiation, and sufficient distance to organs of interest. Organs of interest comprised the brainstem, optic apparatus (optic nerves and chiasm), and cochleae; hippocampi were not included in the optimization protocol. A minimum 20 mm shortest three-dimensional contour-to-contour distance from the PTV to any organ of interest was required to minimize compromises driven by organs of interest and isolate intrinsic PTV-dependent optimization potential. All model plans were created by two medical physicists with more than 15 years of SRS planning experience, adhering to a standardized protocol.

To assess clinical applicability and to calibrate the clinical upper threshold, the model was subsequently evaluated using a set of 98 corresponding routine clinical plans delivered during the study period. The clinical cohort and outlier subgroups are characterized in Supplementary Table S2.

### 2.2 Dose-volume metrics and imaging

Intermediate-dose exposure was quantified using V_10 Gy_, V_12 Gy_, and V_14 Gy_, calculated over the entire body volume. Because the prescription dose was 20 Gy, V_10 Gy_ corresponded to the 50% prescription isodose volume (V_50%_).

The conformity index (CI; defined as the prescription isodose volume divided by the PTV) and the gradient index (GI; defined according to Paddick et al. [12] as V_50%_ divided by V_100%_) were also evaluated. For benchmarking, R_50%_ was defined as V_50%_/PTV. Accordingly, R_50%_ = GI × CI and V_50%_ = R_50%_ × PTV = GI × CI × PTV. These identities were used to convert published index-based benchmark functions into absolute volumetric trajectories.

All patients underwent contrast-enhanced T1-weighted magnetic resonance imaging with a slice thickness of 1 mm before treatment planning, followed by non-contrast planning computed tomography with identical slice thickness. Targets were delineated on magnetic resonance images and transferred to planning computed tomography by rigid registration. Deformable registration was not used. Residual magnetic resonance distortion and rigid-registration uncertainty were acknowledged; the 1 mm isotropic PTV margin was intended to mitigate small systematic deviations [15], [16], [17], [18], [19]. Patients were immobilized using a thermoplastic stereotactic mask (BrainLab AG, Munich, Germany), and set-up variations were verified using ExacTrac X-Ray 6D, ETD 2.0 (BrainLab AG, Munich, Germany).

### 2.3 Treatment planning and technique selection

All treatments were delivered using a TrueBeam STx linear accelerator (Varian Medical Systems, Palo Alto, CA, USA) equipped with an HD120 multileaf collimator (MLC; leaf width 2.5 mm). Photon beams with 6 MV in flattening filter-free (FFF) mode were utilized to achieve higher dose rates up to 1400 monitor units (MU)/min, shorter delivery times, and improved dose conformity [16], [20]. The prescription dose was 20 Gy. Treatment plans were normalized with the 80% isodose line encompassing 100% of the PTV, corresponding to a maximum dose of 25 Gy. Therefore, V_10 Gy_ corresponds to V_50%_.

In patients with multiple brain metastases, a separate plan was generated for each lesion, with the isocenter placed at the geometric center of the individual target. Treatment planning was performed using Eclipse, v15.6.06 with the integrated optimization engine (Photon Optimizer v15.6.06). Dose calculation was conducted using the Analytical Anisotropic Algorithm (AAA) v15.6.06 with a calculation grid size of 0.125 cm. Plans were generated and delivered as volumetric modulated arc therapy (RapidArc) treatments. The standardized planning template consisted of one full arc and three equally spaced non-coplanar half arcs to maximize target conformity and optimize dose fall-off. Dose guidance for organs of interest followed the institutional SRS protocol and are summarized in Supplementary Table S1.

### 2.4 Mathematical modeling strategy

To quantify the nonlinear relationship between target size and intermediate-dose spillage, regression analysis in logarithmic space was performed on the model cohort. This approach modeled a power-law relationship, V_50%_ = A × PTV^B^, under idealized planning conditions. Where A denoted the scaling coefficient and B the volume-scaling exponent (volumes in cm^3^). In addition to the mean regression, a model-based 95th-percentile reference (P95) was derived as V_50%_ = 4.10 × PTV^0.83^.

### 2.5 Derivation of clinical tolerance thresholds

A clinically actionable upper tolerance limit was derived from the clinical cohort using quantile regression at q = 0.90 because the clinical scatter plot showed a visually distinct upper-tail group.

The q = 0.90 boundary was parameterized using a tolerance function, $V_{50\%}^{\text{upper}}$= C + (1 + r) × $V_{50\%}^{\text{Ideal}}$, and approximated by the simplified power-law function V_50%_ = 5 × PTV^0.75^ for routine clinical use.

In routine implementation, V_50%_ was calculated after plan generation and compared with the PTV-specific threshold in addition to standard SRS plan-quality metrics. Plans exceeding the threshold were flagged for structured review of target shape, proximity to organs of interest, prior irradiation, couch/collimator geometry, and dose fall-off behavior. If replanning was performed, the original clinical optimization strategy was retained rather than imposing a prespecified V_50%_ target. Prescription isodose line and normalization were unchanged. Couch and collimator angles could be adjusted when considered beneficial by the planner; dynamic multileaf-collimator leaf sequences and MU distribution were then regenerated by inverse optimization using the original coverage, organs of interest, conformity, and dose-fall-off priorities. No additional non-coplanar arcs were introduced. The benchmark served as an outlier-detection tool rather than a hard cost-function constraint.

### 2.6 Comparative analysis and benchmarking strategy

To contextualize the derived volumetric laws, quantitative benchmarking was performed against established SIMT linear accelerator-based models. The Ballangrud-derived R_50%_ trajectory reported by Desai et al. (R_50%_ ≈ 4.8 × PTV^-0.2^) was converted into an absolute trajectory using V_50%_ = R_50 %_ × PTV, yielding V_50%_ ≈ 4.8 × PTV^0.8^. [13], [14]

The predictive GI model by Ballangrud et al. (GI ≈ 4.0 × PTV^-0.2^) was also analyzed to demonstrate the conformity dependence of index-based predictions, because V_50%_ = GI × PTV × CI [13].

### 2.7 Data analysis

Statistical analyses were performed using IBM SPSS Statistics (v29; IBM Corp., Armonk, NY, USA). Graphical visualizations were created using Python (Anaconda Distribution, Jupyter Notebook) with the matplotlib and numpy libraries. The Pearson correlation coefficient (r) and the coefficient of determination (R^2^) were calculated in the log-log space to assess the strength of the relationship. Statistical significance was defined as *p* < 0.05. For interpretation of effect size, |r| values of approximately 0.1, 0.3, and 0.5 were considered small, moderate, and large, respectively.

## 3 Results

The V_10 Gy_, V_12 Gy_, and V_14 Gy_ volumes showed strong and consistent correlations with PTV in log-log space in the model cohort (all *r* > 0.99, *p* < 0.001). Although V_14 Gy_ showed a slightly higher correlation, V_10 Gy_ was selected as the primary parameter because it corresponded to the V_50%_ volume in the 20 Gy prescription protocol. These relationships are shown in Fig. 1. The relative indices CI and GI showed large negative correlations with PTV (CI: *r* = −0.55; GI: *r* = −0.81; both *p* < 0.001). The final model function was derived from the model cohort (*n* = 178) by back-transformation from log-log regression (R^2^ = 0.992). The resulting ideal mean model was $V_{50\%}=3.71\times {\mathrm{PTV}}^{0.83}$ and the conservative P95 reference was V_50%_ ≤ 4.10 × PTV^0.83^ (Fig. 2B). The ideal mean model, P95 reference, clinical threshold, and converted multi-target benchmark are shown in Fig. 2. In the clinical cohort (*n* = 98), regression confirmed robust volume scaling (R^2^ = 0.92). The clinical upper threshold was V_50%_ = 5 × PTV^0.75^ (Table 1). In total, 88 of 98 clinical plans (90%) were below this threshold, while 10 of 98 plans (10%) exceeded it. The plans exceeding the threshold occurred across a broad PTV range (0.34–3.59 cm^3^).

**Fig. 1:**
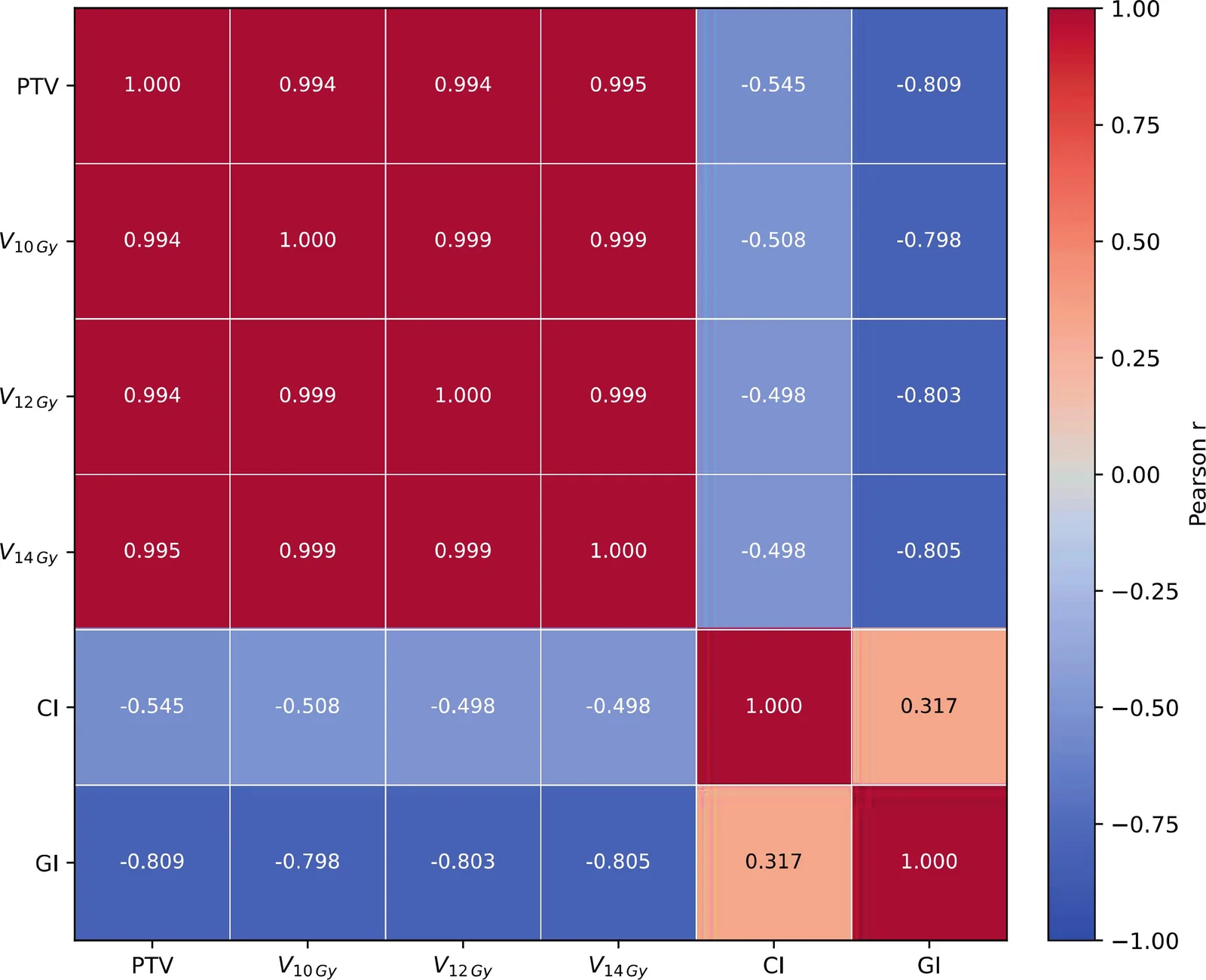
Pearson correlation matrix between planning target volume, V_10 Gy_, V_12 Gy_, V_14 Gy_, conformity index, and gradient index in the model cohort. Colour scale indicates r values. All *p* < 0.001.

**Fig. 2:**
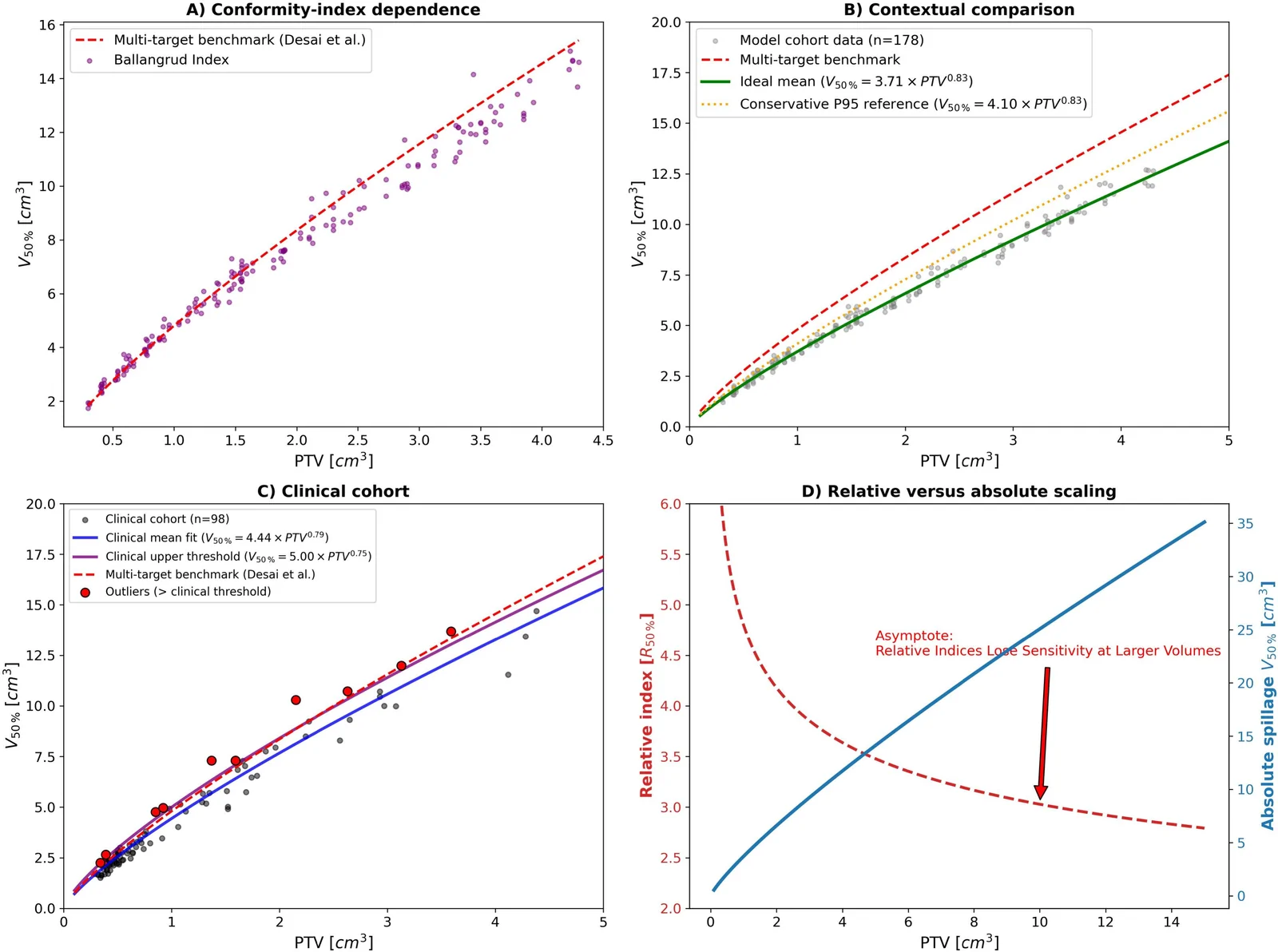
Volume-specific V_50%_ benchmarks in the model and clinical cohorts. A, effect of conformity-index variation on index-based predictions. B, model cohort with ideal mean and conservative P95 reference. C, clinical cohort with q = 0.90 upper threshold and outliers. D, comparison of relative R_50%_ and absolute V_50%_ scaling.

**Table 1:** Ideal P95 reference and clinical q = 0.90 upper threshold for V_50%_ by planning target volume.

PTV (cm^**3**^)	V_50%_ (cm^**3**^) (4.1 × PTV^0.83^)	V_50%_ (cm^**3**^) (5 × PTV^0.75^)		PTV (cm^**3**^)	V_50%_ (cm^**3**^) (4.1 × PTV^0.83^)	V_50%_ (cm^**3**^) (5 × PTV^0.75^)
0.3	1.51	2.03		2.4	8.48	9.64
0.4	1.92	2.51		2.5	8.77	9.94
0.5	2.31	2.97		2.6	9.06	10.24
0.6	2.68	3.41		2.7	9.35	10.53
0.7	3.05	3.83		2.8	9.64	10.82
0.8	3.41	4.23		2.9	9.92	11.11
0.9	3.76	4.62		3.0	10.20	11.40
1.0	4.10	5.00		3.1	10.49	11.68
1.1	4.44	5.37		3.2	10.77	11.96
1.2	4.77	5.73		3.3	11.04	12.24
1.3	5.10	6.09		3.4	11.32	12.52
1.4	5.42	6.44		3.5	11.60	12.79
1.5	5.74	6.78		3.6	11.87	13.07
1.6	6.06	7.11		3.7	12.14	13.34
1.7	6.37	7.44		3.8	12.42	13.61
1.8	6.68	7.77		3.9	12.69	13.88
1.9	6.98	8.09		4.0	12.96	14.14
2.0	7.29	8.41		4.1	13.22	14.41
2.1	7.59	8.72		4.2	13.49	14.67
2.2	7.89	9.03		4.3	13.76	14.93
2.3	8.18	9.34				

Ideal P95 reference and clinical upper threshold for V_50%_ by planning target volume. Values are given in cm^3^.

Among the 10 plans exceeding the threshold, three were considered clinically justified because of pronounced target asymmetry (*n* = 2) or proximity to an area of previous irradiation (*n* = 1). The remaining seven plans lacked anatomical justification and underwent targeted replanning. All seven replanned cases satisfied the clinical upper threshold, with a median V_50%_ reduction of 14% (range 7–29%) and preserved target coverage (See Fig. 3). Plans exceeding the threshold and replanning changes are summarized in Supplementary Table S3. A representative before-and-after dose-distribution example is provided in Supplementary Fig. S1.

**Fig. 3:**
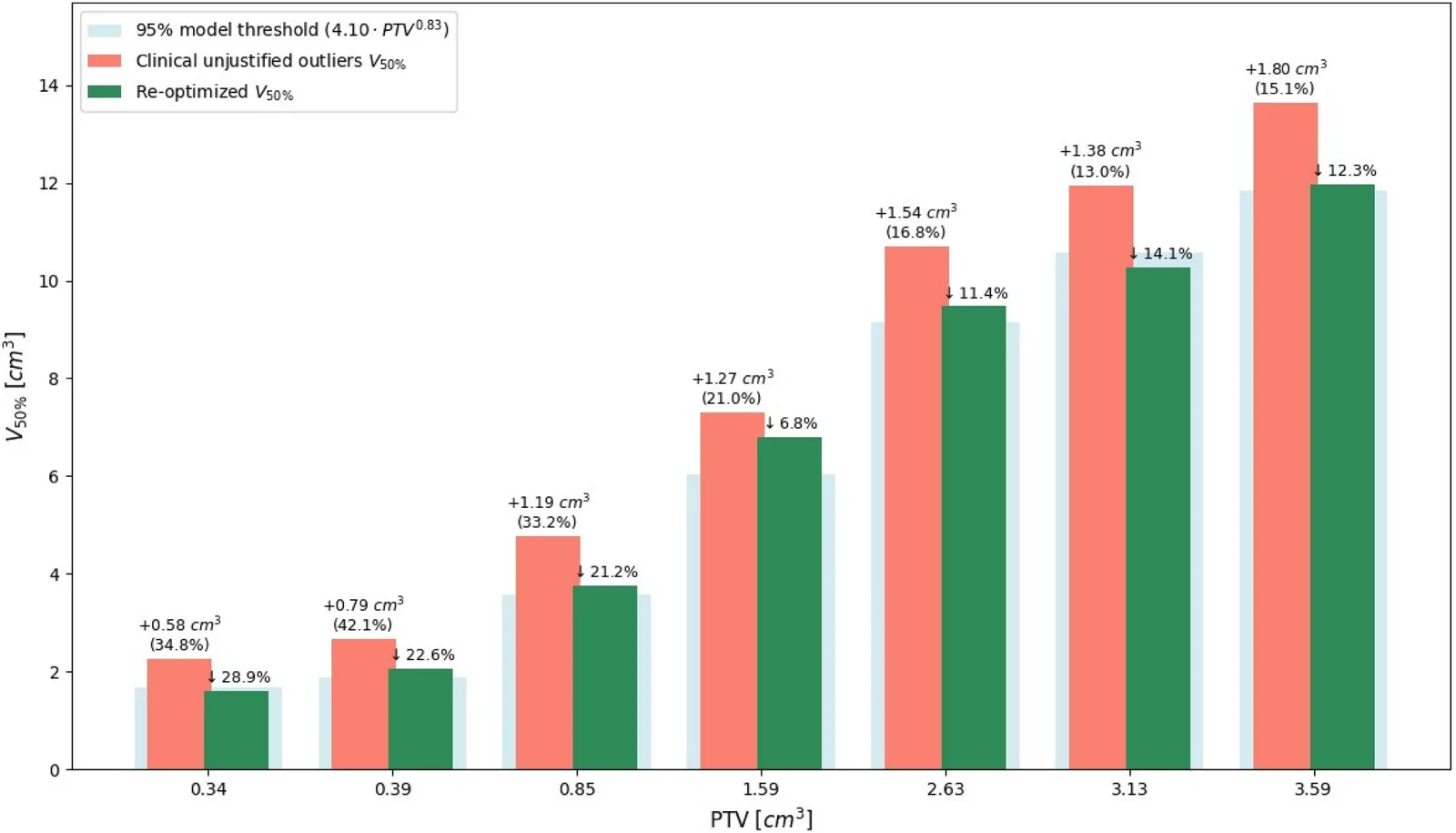
Original and replanned V_50%_ values for seven threshold-exceeding plans without clinical justification. Bars show original clinical plans, replanned plans, and the conservative P95 reference. Labels indicate excess above the reference and percentage reduction after replanning.

## 4 Discussion

This study validated a simple, volume-based model for estimating absolute intermediate-dose spillage (V_50%_; equivalent to V_10 Gy_ for 20 Gy prescriptions) in linear accelerator-based single-target SRS for brain metastases. Due to its simplicity and volume-only input, the proposed model supported rapid plan evaluation and provided a standardized framework for benchmarking treatment planning across institutions with comparable technology. Beyond routine quality assurance (QA), the established reference standards (V_ideal_ vs. V_clinical_) enabled quantitative assessment of plan quality against physical potential rather than fixed adverse-event limits.

Based on 276 treatment plans, an ideal P95 reference and a clinical q = 0.90 upper threshold were derived. Unlike Ballangrud/Desai mean regressions, which approximately half of plans exceed by design, the q = 0.90 threshold was intended as an upper-tail review trigger [13], [14]. The benefit of lower triggers was not tested and should be investigated, particularly for larger lesions and with outcome-based or normal-tissue-complication-probability modeling, whereas the reduced exponent (B = 0.75) avoided excessive tolerance expansion at larger PTVs.

The use of a power-law function aligned with the geometric principle that dose spillage is primarily driven by surface area, as consistently described by Desai et al. [9]. However, a direct comparison was limited by two factors. First, the present analysis was strictly restricted to single lesions, whereas the published multi-target trajectories described mean behavior in multiple-lesion planning scenarios [13], [14]. Second, the benchmark reported by Desai et al. represented a population mean [14], whereas the derived thresholds defined upper tolerance limits (q = 0.90 for clinical data and P95 for the ideal model). Published multi-target benchmark trajectories are valuable empirical references, but they primarily describe volume- or index-based mean behavior and do not explicitly include lesion number, inter-target distance, lesion location, or dose bridging as independent predictors in the benchmark equations. Therefore, a single-target baseline is required before the additional intermediate-dose burden of simultaneous multiple-target planning can be quantified.

Regarding clinical safety standards, the results addressed the lack of continuous optimization thresholds in current guidelines. While fixed limits (e.g., Minniti's V_12 Gy_ < 10 cm^3^) correctly identified high-risk scenarios for radionecrosis, they are unsuitable for enforcing the as-low-as-reasonably-achievable (ALARA) principle for smaller tumors [7]. The volume-dependent model operationalized ALARA by translating the abstract requirement for minimization into a quantifiable physical reference. This ensured that plan quality was measured against the individual optimization potential of the PTV rather than a rigid ceiling. This is relevant for salvage SRS and reirradiation, where avoidable intermediate-dose spill in an initial plan may increase cumulative normal-brain dose exposure and restrict subsequent treatment options.

The practical impact of this model was demonstrated through targeted replanning of outliers. The median V_50%_ reduction was 14% (range: 7–29%) without compromising target coverage. Clinically, the threshold was used after plan generation and before final approval as a review trigger, not as a hard constraint. This does not mean that V_50%_ or dose-fall-off objectives should be absent from optimization; fall-off objectives remained part of routine VMAT planning. The model's value was to reveal cases in which the spill remained unexpectedly high after routine optimization, prompting review of couch/collimator geometry and the inverse-optimized multileaf-collimator leaf sequence and monitor-unit distribution. Published VMAT/SRS studies showed that prescription-isodose selection and hot-spot penalties could influence plan quality [21], [22], [23]; such strategies and hard V_50%_ objectives should be validated prospectively because they may alter coverage, conformity, organ sparing, or low-dose bath.

Methodologically, the proposed benchmark favored absolute spillage volume as a complementary metric over relative indices (GI, R_50%_), for which clear, volume-specific thresholds in SIST are often lacking. Conformity and gradient indices remain established metrics, and optimization of conformity and gradient is already standard in SRS [15]. However, relative indices can be affected by conformity and prescription-isodose effects and cannot be directly summed across lesions. Since radionecrosis risk is driven by the absolute volume of intermediate dose, the absolute V_50%_ model aligned more strictly with dose guidance for adverse events [6]. Furthermore, unlike relative indices, absolute volumes can be meaningfully summed across multiple targets, making this metric inherently scalable for cumulative side-effect assessment in future multi-metastasis applications and retreatment settings.

The study had several limitations. It was performed at a single institution using one treatment platform and calculation algorithm (TrueBeam STx, HD120 MLC, 6 MV, FFF, AAA); benchmarking should therefore be restricted to comparable technology and performed only after local validation. The selected V_10 Gy_ to V_14 Gy_ dose levels are most directly linked to adverse events in the normal brain, and the model does not define tissue-specific dose guidance for non-brain structures. Target shape may affect achievable spillage; irregular volumes such as vestibular schwannoma require separate validation, and the target asymmetry index (Qasym) may help distinguish geometry-driven threshold crossings from technical outliers [24]. The V_50%_ samples one intermediate-dose level; improvements should therefore be reviewed together with coverage, conformity, gradient, dose guidance for organs of interest, and the low-dose bath. Future work should extend the quantile-based approach to multi-target planning.

In conclusion, by defining an ideal physical reference standard and a statistically derived clinical upper tolerance limit ($V_{50\%}^{\text{upper}}\leq 5\times {\mathrm{PTV}}^{0.75}$), this study established a volume-based benchmark for absolute intermediate-dose spillage in linear accelerator-based single-target SRS for brain metastases. The benchmark can identify plans that warrant structured review and may prevent avoidable dose spill when no anatomical justification exists. It should be applied as a complementary quality-assurance reference together with target coverage, dose guidance for organs of interest, conformity, gradient, and low-dose metrics.

## CRediT authorship contribution statement

**Youness Nour:** Writing – original draft, Visualization, Software, Methodology, Investigation, Formal analysis, Data curation, Conceptualization. **Shari Wiegreffe:** Writing – review & editing, Conceptualization, Data curation, Validation. **Tuba Tekin:** Writing – review & editing, Conceptualization, Data curation, Validation. **Lara Caglayan:** Writing – review & editing, Data curation, Formal analysis. **Christina Leitzen:** Writing – review & editing, Conceptualization, Formal analysis. **Cas S. Dejonckheere:** Writing – review & editing, Conceptualization, Formal analysis. **Davide Scafa:** Writing – review & editing, Conceptualization, Formal analysis. **Julian P. Layer:** Writing – review & editing, Conceptualization, Formal analysis. **Victoria Volkenborn:** Writing – review & editing, Conceptualization, Formal analysis. **Fabian Kugel:** Writing – review & editing, Conceptualization, Formal analysis. **Stephan Garbe:** Writing – review & editing, Conceptualization, Formal analysis. **Christos Moustakis:** Writing – review & editing, Conceptualization, Formal analysis. **Dimos Baltas:** Writing – review & editing, Conceptualization, Formal analysis, Methodology. **Gustavo R. Sarria:** Writing – review & editing, Conceptualization, Formal analysis. **Anca-L. Grosu:** Writing – review & editing, Conceptualization, Formal analysis. **Eleni Gkika:** Writing – review & editing, Supervision, Conceptualization, Methodology.

## Ethics declaration

The authors have NOT obtained informed consent from participants or their legal representatives. Individual informed consent was not obtained. The approved protocol (Ethics Committee of the Medical Faculty of the University of Bonn covered retrospective analysis of pseudonymized routine treatment and planning data without individual consent. No additional interventions or patient contact occurred, and no identifiable patient information is reported.

This study was performed in compliance with relevant laws, regulatory frameworks and guidelines where the research took place. This study was approved by the The Ethics Committee of the Medical Faculty of the University of Bonn.

(Approval No. 2024–105-BO)

## Declaration of generative AI and AI-assisted technologies in the writing process

During the preparation of this work, the authors used ChatGPT (OpenAI) for language editing and translation support. After using this tool, the authors reviewed, edited, and verified the content as needed and take full responsibility for the content of the publication.

## Funding

This research did not receive any specific grant from funding agencies in the public, commercial, or not-for-profit sectors.

## Declaration of competing interest

The authors declare that they have no known competing financial interests or personal relationships that could have appeared to influence the work reported in this paper.

## Appendix A Supplementary data

Supplementary material: Supplementary Tables S1–S3: institutional dose guidance, cohort characteristics, and original versus replanned dose-volume metrics. Supplementary Fig. S1: representative dose distributions before and after replanning.
