# Supplementary material for: Benchmarking intermediate dose spillage in single target stereotactic radiosurgery for brain metastases
Source: Phys Imaging Radiat Oncol. 2026 Oct 9;41:101105. doi: 10.1016/j.phro.2026.101105 (PMC13682193; doi:10.1016/j.phro.2026.101105)
Supplement: Supplementary file 1 [file mmc1.docx]

**Supplementary material**

**Benchmarking intermediate dose spillage in single target stereotactic radiosurgery for brain metastases**

**Table S1. Institutional planning objectives and dose guidance for organs of interest.**

| Organ of interest / objective | 1 fraction | Comment |
| --- | --- | --- |
| Target coverage / normalization | 20 Gy prescription; 80% isodose line; V100% typically 99.5-100% of PTV | Planning aim; normalization was not changed during replanning. |
| Optic nerves / optic chiasm | D0.2 cm³ < 8 Gy; Dmax 10 Gy | Dose guidance from SABR Consortium / Timmermann constraint list. |
| Cochleae | Dmean < 4 Gy; Dmax 9 Gy | Used when clinically relevant. |
| Brainstem | D0.1 cm³ < 10 Gy; Dmax 15 Gy | High-priority organ of interest. |
| Spinal cord including medulla | D0.1 cm³ < 10 Gy; Dmax 14 Gy | Applied if within relevant treatment field. |
| Brain | V12 Gy < 5 cm³ | Used as normal-brain dose guidance, not as a replacement for plan-quality benchmarking. |
| Lens | D0.1 cm³ < 1.5 Gy | Applied when close to treatment field. |
| Orbit / orbital contents | D0.1 cm³ < 8 Gy | institutional dose-guidance |
| Hippocampi | No hard constraint applied | Not included in the optimization protocol for this cohort. |

Abbreviations: Dmax, maximum point dose; PTV, planning target volume; Vx Gy, volume receiving at least x Gy. The listed values were used as institutional dose guidance and planning objectives; clinical prioritization depended on target coverage and organ-of-interest proximity.

Source note: Optic-pathway, cochlear, brainstem, spinal-cord, lens, and orbital dose guidance was informed by [1,2] and adapted for institutional planning. Target coverage, brain V12 Gy, and hippocampal entries reflect local objectives.

**References for Table S1**

[1] UK SABR Consortium. Stereotactic Ablative Body Radiotherapy (SABR): A Resource. Version 6.1. 2019. Appendix A, p. 121. https://www.sabr.org.uk/wp-content/uploads/2019/04/SABRconsortium-guidelines-2019-v6.1.0.pdf

[2] Timmerman R. A story of hypofractionation and the table on the wall. Int J Radiat Oncol Biol Phys. 2022;112(1):4–21. doi:10.1016/j.ijrobp.2021.09.027.

**Table S2. Cohort characteristics derived from the model and clinical dose-volume exports.**

| Characteristic | Model cohort | Clinical cohort | Threshold-exceeding plans | Replanned unjustified outliers |
| --- | --- | --- | --- | --- |
| Number of lesions/plans | 178 | 98 | 10 | 7 |
| PTV, median (IQR), cm³ | 1.55 (0.88-2.88) | 0.64 (0.43-1.52) | 1.48 (0.87-2.51) | 1.59 (0.62-2.88) |
| PTV, range, cm³ | 0.30-4.30 | 0.30-4.38 | 0.34-3.59 | 0.34-3.59 |
| V50%, median (IQR), cm³ | 5.60 (3.35-8.68) | 2.97 (2.27-5.79) | 7.30 (4.82-10.61) | 7.30 (3.72-11.36) |
| V50%, range, cm³ | 1.21-12.70 | 1.52-14.70 | 2.25-13.69 | 2.25-13.69 |
| R50%, median (IQR) | 3.43 (3.12-3.81) | 4.60 (4.06-5.21) | 5.06 (4.20-5.56) | 4.59 (3.95-6.11) |
| Prior irradiation | Excluded by design | 17 | 1 clinically justified threshold exceedance | 0 |
| Distance to organs of interest | ≥ 20 mm by design | Not restricted; assessed clinically | Reviewed case by case | No anatomical justification identified |
| Supratentorial lesions, n (%) | 144 (81%) | 76 (78%) | 8 (80%) | 6 (86%) |
| Infratentorial lesions, n (%) | 34 (19%) | 22 (22%) | 2 (20%) | 1 (14%) |
| Skull-base / deep lesions, n (%) | 0 (0%) | 0 (0%) | 0 (0%) | 0 (0%) |

**Table S3. Threshold exceedances and replanning results.**

| Case | PTV (cm³) | Qasym | Clinical upper threshold (cm³) | Excess above upper threshold (cm³) | Ideal P95 reference (cm³) | Status / reason | Replanned V50% (cm³) | Reduction (%) |
| --- | --- | --- | --- | --- | --- | --- | --- | --- |
| 1 | 0.34 | 1.19 | 2.23 | 0.02 | 1.67 | Replanned; no anatomical justification | 1.60 | 29 |
| 2 | 0.39 | 1.03 | 2.47 | 0.19 | 1.88 | Replanned; no anatomical justification | 2.06 | 23 |
| 3 | 0.85 | 1.34 | 4.43 | 0.34 | 3.58 | Replanned; no anatomical justification | 3.76 | 21 |
| 4 | 0.92 | 1.09 | 4.70 | 0.27 | 3.83 | Clinically justified; prior irradiation | — | — |
| 5 | 1.37 | 1.84 | 6.33 | 0.97 | 5.32 | Clinically justified; asymmetry | — | — |
| 6 | 1.59 | 1.39 | 7.08 | 0.22 | 6.02 | Replanned; no anatomical justification | 6.80 | 7 |
| 7 | 2.15 | 1.69 | 8.88 | 1.41 | 7.74 | Clinically justified; asymmetry | — | — |
| 8 | 2.63 | 1.24 | 10.33 | 0.39 | 9.15 | Replanned; no anatomical justification | 9.50 | 11 |
| 9 | 3.13 | 1.38 | 11.77 | 0.22 | 10.57 | Replanned; no anatomical justification | 10.30 | 14 |
| 10 | 3.59 | 1.09 | 13.04 | 0.65 | 11.84 | Replanned; no anatomical justification | 12.01 | 12 |

**Table S3 continued. Coverage and complementary metrics for the seven replanned outliers.**

| Case | PTV (cm^3^) | Qasym | D98% original / replanned (Gy) | D99% original / replanned (Gy) | V100% original / replanned (cm^3^) | V12 Gy original / replanned (cm^3^) | V5 Gy original / replanned (cm^3^) | CI original / replanned | GI original / replanned |
| --- | --- | --- | --- | --- | --- | --- | --- | --- | --- |
| 1 | 0.34 | 1.19 | 22.73/22.65 | 20.64/20.52 | 0.49/0.45 | 1.64/1.52 | 7.02/6.83 | 1.44/1.32 | 4.59/3.56 |
| 2 | 0.39 | 1.03 | 21.3/21.05 | 21.13/20.81 | 0.62/0.56 | 2.00/1.62 | 8.02/6.05 | 1.59/1.44 | 4.29/3.67 |
| 3 | 0.85 | 1.34 | 21.31/21 | 21.09/20.83 | 1.23/1.15 | 3.52/2.92 | 15.68/11.98 | 1.45/1.35 | 3.88/3.27 |
| 6 | 1.59 | 1.39 | 21.23/21.32 | 21.02/21.11 | 2.10/2.13 | 6.63/5.44 | 22.32/19.30 | 1.32/1.34 | 3.48/3.19 |
| 8 | 2.63 | 1.24 | 21.41/21.35 | 21.31/21.14 | 3.48/3.35 | 8.21/7.61 | 31.92/28.70 | 1.32/1.27 | 3.07/2.84 |
| 9 | 3.13 | 1.38 | 21.39/21.26 | 21.12/21.03 | 3.88/3.90 | 9.33/9.01 | 33.42/31.80 | 1.24/1.25 | 3.09/2.64 |
| 10 | 3.59 | 1.09 | 22.11/22.14 | 21.93/21.87 | 5.42/4.81 | 10.87/10.02 | 32.90/34.50 | 1.51/1.34 | 2.53/2.49 |

Supplementary Fig. S1. Representative dose distributions for case 9 before and after replanning.

Original-plan views are shown in the left column and replanned views in the right column; axial, coronal, and sagittal planes are displayed. Replanning for this threshold-exceeding outlier included a 60° change in couch rotation, because this was considered dosimetrically beneficial by the planner.

| **A. Original plan** | **B. Replanned plan** |
| --- | --- |
|  |  |

The replanned distribution illustrates the reduced intermediate-dose spillage while preserving target coverage.
